# YIP1 family member 4 (YIPF4) is a novel cellular binding partner of the papillomavirus E5 proteins

**DOI:** 10.1038/srep12523

**Published:** 2015-08-03

**Authors:** Marietta Müller, Christopher W. Wasson, Ramya Bhatia, Sally Boxall, David Millan, Grace Y.S. Goh, Jürgen Haas, Nicola J. Stonehouse, Andrew Macdonald

**Affiliations:** 1 University of Leeds, UK; 2 Division of Pathway Medicine, The University of Edinburgh, UK; 3 Department of Pathology, Southern General Hospital, Glasgow, Scotland, UK

## Abstract

E5 proteins are amongst the least understood of the Human Papillomavirus (HPV) encoded gene products. They are small, membrane-integrated proteins known to modulate a number of critical host pathways associated with pathogenesis including growth factor receptor signaling and immune evasion. Their role in the virus life cycle is less clear, indicating a role in the productive stages of the life cycle. However, a mechanism for this is currently lacking. Here we describe the identification of a novel binding partner of E5, YIPF4 using yeast two-hybrid analysis. YIPF4 is also a poorly characterized membrane spanning protein. Mutagenesis studies implicated the transmembrane regions of each protein as important for their interaction. Binding to YIPF4 was found for all E5 proteins tested suggesting that this interaction may mediate a conserved E5 function. In normal human keratinocytes YIPF4 expression was down-regulated upon differentiation and this reduction was partially rescued in cells harbouring HPV. Despite the conserved nature of the interaction with E5, siRNA mediated depletion of YIPF4 failed to impede two well-characterized functions of E5, namely EGFR trafficking or HLA class I presentation. Continued studies of YIPF4 are warranted to determine its role in the PV life cycle.

Human papillomaviruses (HPVs) are small, double-stranded, non-enveloped DNA viruses. More than 170 different HPV types have been isolated and sub-divided into different genera of which the α-genus is clinically most relevant. This genus can be categorized into high-risk (e.g. HPV16, 18, 31), low-risk (e.g. HPV6, 11, 83) or cutaneous (e.g. HPV2) HPV types. High-risk HPV types are associated with 99.7% of cervical cancer cases with HPV16 and HPV18 being the most prevalent in this type of cancer^1^. Low-risk HPV types are the causative agent of benign mucosal growths including genital warts or recurrent respiratory papillomatosis^2^ while the cutaneous HPV types cause common skin warts^3^. HPVs infect the basal cells of skin and mucosal epithelia and their life cycle is absolutely dependent on keratinocyte differentiation^4^. They express three oncogenic proteins: E6, E7 and E5. E6 and E7 are well characterized and drive suprabasal cells into S-phase by inhibiting tumor suppressor pathways (p53 and retinoblastoma protein, respectively) to allow replication of the viral genome.

HPV16 E5 (16E5) is a small hydrophobic protein consisting of 83 amino acids, which forms three putative trans-membrane α-helices and appears to multimerize into a hexameric viroporin^5^,^6^,^7^,^8^. It is the least understood of the three HPV oncoproteins and most information about it is derived from overexpression systems due to a lack of antibody detection reagents. The majority of 16E5 localizes to the endoplasmic reticulum (ER), the Golgi apparatus (GA), the nuclear envelope and to a lesser extent the plasma membrane^9^,^10^,^11^. The oncogenic properties of 16E5 are likely based on manipulation of the epidermal growth factor receptor (EGFR) and its downstream signaling, however, the exact mechanisms are controversial. Several publications report an interaction of 16E5 with the 16K subunit of the vacuolar H^+^-ATPase as cause for decreased endosomal acidification, promoting EGFR recycling and thus constitutive signaling^12^,^13^. Other groups have shown an effect of 16E5 on the endocytic pathway. 16E5 prevents fusion of endosomes to lysosomes promoting reduced compartment acidification and increased EGFR recycling^14^. The interaction partners involved in endocytic trafficking are still to be determined.

E5 also contributes to viral immune evasion. Cells expressing a number of E5 proteins impair the expression of HLA class I molecules^15^,^16^, but again the precise mechanisms remain to be elucidated. E5 is also involved in the HPV lifecycle and has been shown to impact on keratinocyte proliferation and control of virus genome amplification^17^,^18^.

A yeast two-hybrid (Y2H) screen was used to identify the GA resident transmembrane protein, YIPF4, as a novel interaction partner of 16E5. We confirmed this interaction in cervical cells and mapped the regions of each protein required to mediate binding. We also demonstrated YIPF4 expression in differentiating primary keratinocytes and clinical samples of cervical intraepithelial neoplasia. YIPF4 was a binding partner common to all E5 proteins tested, suggesting a conserved function of the interaction. We were not able to demonstrate an effect of depleting cells of YIPF4 on EGFR trafficking or HLA class I presentation, two key cellular processes perturbed by E5. These data suggest that YIPF4 may be involved in an uncharacterized aspect of E5 biology.

## Results

### A Y2H screen identifies YIPF4 as a novel target of 16E5

A semi-automated Y2H assay was used to identify new potential interaction partners of 16E5. 16E5, as bait, was expressed as a fusion to the C-terminus of the DNA binding domain of GAL4. This was screened against a prey library of C-terminal fusion proteins to the GAL4 activation domain from HeLa cells as well as human testis epithelial cells. Positive single colonies were isolated and identified by PCR amplification. A single positive colony was confirmed to encode the full length sequence of a protein termed YIPF4 (data not shown). YIPF4 is a poorly characterized Golgi-apparatus (GA) localized protein with a potential role in ER to GA trafficking^19^.

### 16E5 and YIPF4 localize in membranous cell compartments

**Figure 1:**
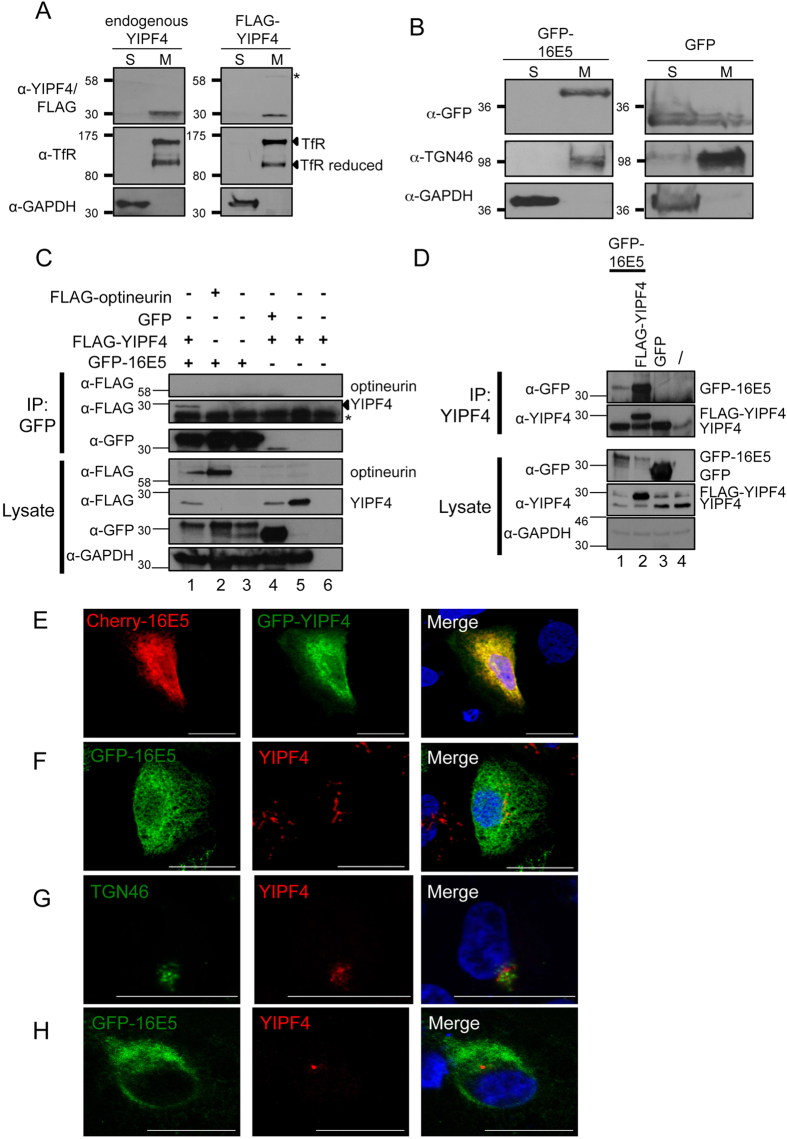
YIPF4 is a novel HPV16 E5 interacting protein. YIPF4 associates with cytoplasmic membranes. **A**) HEK293T cells were mock treated or transfected with a FLAG-YIPF4 plasmid and fractionated into soluble cytoplasmic (marker protein GAPDH) (S) and membraneous cytoplasmic (marker protein transferrin receptor) (M) fractions and probed with antibodies against YIPF4 and FLAG. *possible YIPF4 dimer. **B**) Cells were transfected with GFP-16E5 or GFP alone and fractionated using the same method. TGN46 was utilised as the membrane marker protein. **C**) Co-immunoprecipitations (Co-IP) demonstrated an interaction between GFP-16E5 and FLAG-YIPF4. Lysates containing FLAG-YIPF4 and GFP-16E5 as well as the negative controls GFP and FLAG-optineurin were precipitated with a GFP antibody coupled to beads. Precipitates were probed with antibodies against GFP and the FLAG epitope. Lane 6 is a beads only control. *non-specific band. All gels were run under the same experimental conditions. **D**) 16E5 binds to endogenously expressed YIPF4. GFP-16E5, GFP (negative control) and FLAG-YIPF4 (positive control) lysates were precipitated with a YIPF4 antibody to pulldown endogenous YIPF4. ‘ / ’ = cell lysate without overexpressed proteins incubated with uncoupled beads. Marker in kDa. **E**) SiHa cells over-expressing GFP-YIPF4 and mCherry-16E5 were fixed and imaged using a Zeiss LSM700 confocal microscope. **F**) Cells overexpressing a GFP-16E5 fusion were stained with an antibody raised against YIPF4. **G**) HPV negative C33A cells were stained for endogenous YIPF4 and the Golgi apparatus marker TGN46. **H**) C33A cells transfected with GFP-16E5 were stained for endogenous YIPF4. In all experiments cell nuclei were visualised with DAPI (blue). Scale bars = 20 μm.

16E5 is known to be a trans-membrane protein with putative ER and GA localization^8^. By comparison, despite the hydrophobic nature of YIPF4, there is little experimental evidence for its predicted membrane association^19^. We first set out to confirm the membrane association of both proteins. For this, cells expressing FLAG epitope tagged and endogenous YIPF4 were partitioned into soluble and membrane protein fractions. Fractions were analyzed by western blot and their identity confirmed using established marker proteins (Fig. 1A–B). Endogenous YIPF4 was clearly present in the membranous fraction and addition of a FLAG epitope did not alter the localization (Fig. 1A). Lack of specific antibodies recognizing 16E5 necessitated the use of an epitope tagged E5 protein in this study. GFP-16E5 was also exclusively found within the membranous fraction (Fig. 1B). By comparison, an isolated GFP control was found mainly in the soluble fraction, demonstrating that the fluorescent tag was not responsible for the observed localization of E5 (Fig. 1B).

### 16E5 and YIPF4 interact in mammalian cells

Lysates from HEK293T cells expressing a GFP-16E5 fusion protein and FLAG-YIPF4 were subject to Co-IP with a GFP antibody (Fig. 1C). Western blot confirmed the successful precipitation of FLAG-YIPF4 by GFP-16E5 (lane 1). Negative controls demonstrated that the interaction was not mediated by either epitope tag (lanes 2–5) (use of a FLAG-tagged cellular protein optineurin^20^ and GFP alone) or the antibody-bead matrix (lane 6). To confirm that 16E5 could interact with endogenous YIPF4 protein in a physiologically relevant cell line, lysates from the cervical cell line C33A expressing GFP-16E5 alone or co-expressed with FLAG-YIPF4 were precipitated using an antibody against YIPF4 (Fig. 1D). Endogenous YIPF4 successfully precipitated 16E5 (lane 1), although at lower levels compared to the exogenously expressed FLAG-YIPF4 (lane 2). GFP alone failed to interact with endogenous YIPF4 (lane 3). A low level of endogenous YIPF4 non-specifically bound to the uncomplexed bead matrix (lane 4), however, this did not affect the observation that physiological levels of YIPF4 can interact with 16E5. We also sought to further investigate the localization of over-expressed YIPF4 and determine whether it was found in similar regions of the cell as 16E5. Initially for these experiments SiHa cells were transfected with expression plasmids encoding mCherry-16E5 and GFP-YIPF4 and the cellular localization assessed by confocal microscopy. SiHa cells provided a model cell lines also expressing the major HPV oncoproteins E6 and E7 and as such may represent an environment found during natural infection. Both proteins exhibited cytoplasmic staining and a high degree of co-localization (Fig. 1E). In contrast, endogenous YIPF4 displayed a more restricted localization, reminiscent of the Golgi, with limited co-localization in SiHa cells (Fig. 1F). To control against any impact of E6 and E7 expression on YIPF4 localization, the HPV negative cervical cancer cell line C33A was stained for endogenous YIPF4. In C33A cells YIPF4 also displayed a restricted localization which did not appear to overlap with either a marker of the trans-Golgi (TGN46) or GFP-16E5 staining (Fig. 1G–H).

### YIPF4 interacts with E5 from a representative panel of α-genus HPV types

**Figure 2:**
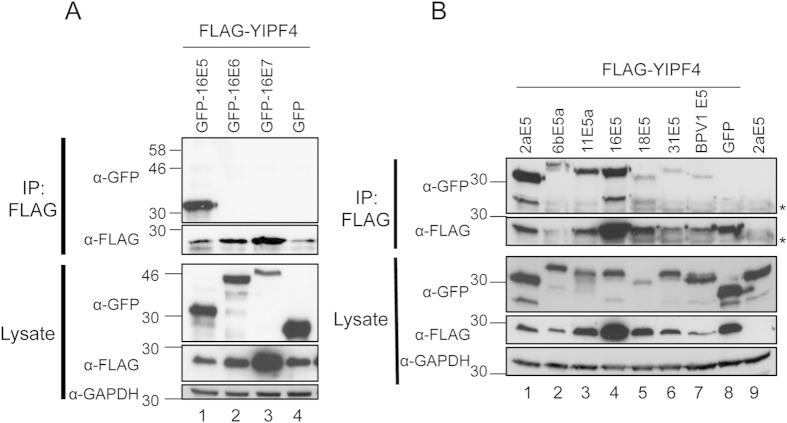
YIPF4 interacts with E5 from a representative panel of PV types. **A**) YIPF4 binds to E5 but not other HPV16 expressed oncoproteins. HEK239T cell lysates containing GFP fusions of the three HPV16 oncoproteins were precipitated using FLAG beads. Eluates were probed with antibodies recognising GFP and the FLAG epitope. **B**) YIPF4 binds to a panel of PV encoded E5 proteins. PV encoded E5 proteins (cutaneous = 2a, low-risk = 6b, 11, high-risk = 16, 18, 31, ungulate = BPV1) were co-expressed as GFP fusion proteins with FLAG-YIPF4. FLAG beads were used to precipitate YIPF4 binding proteins. All gels were run under the same experimental conditions. Representative western blots are shown. Marker in kDa. ‘ * ’ = antibody light chain.

We next sought to determine whether YIPF4 was capable of interacting with the other HPV16 encoded oncoproteins. For this, FLAG-YIPF4 was used as bait and GFP fusion proteins of 16E5 (positive control), 16E6 and 16E7 as prey. Western blot confirmed the interaction between FLAG-YIPF4 and GFP-16E5 (Fig. 2A lane 1). By contrast, no interaction was observed between FLAG-YIPF4 and 16E6, 16E7 or GFP alone (lanes 2–4). YIPF4 appears to be an E5-specific binding partner.

Whilst the amino acid sequences of E5 proteins are divergent, they do share a hydrophobic nature. Although studies of E5 proteins from PV types other than HPV16 are limited, they have demonstrated conserved interactions with a small number of host targets^21^. We investigated whether E5 from different PV types were able to interact with YIPF4. Co-IPs were performed with representative E5 proteins from α-PVs (cutaneous HPV2a, low-risk HPV 6b, 11, high-risk HPV16, 18, 31) and the ungulate BPV1 of the δ-PVs. FLAG-YIPF4 was used as bait and GFP fusion E5 proteins as prey (Fig. 2B). All E5 proteins were precipitated indicating that the interaction is widely conserved amongst PV E5 proteins despite their poor sequence identity.

### Binding of 16E5 to YIPF4 is mediated by the trans-membrane domains of each protein

**Figure 3:**
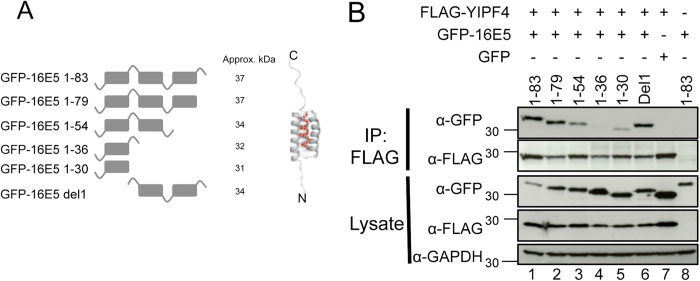
Mapping the regions of 16E5 necessary for binding to YIPF4. **A**) Schematic representation of 16E5 truncation mutants generated as GFP fusion proteins, based on previous published work^6^,^33^. The designated name for each truncation mutant is indicated on the left. The del1 truncation mutant forms an exception and consists of the first predicted transmembrane domain (TMD) only. Boxes indicate TMD and loops luminal (subscript) and cytosolic (superscript) sites. **B**) Lysates containing GFP-16E5 truncation mutants and FLAG-YIPF4 were precipitated with FLAG beads and probed with antibodies against GFP and FLAG. Lysates were also probed with an antibody against GAPDH to demonstrate equal loading. GFP alone served as a negative control. All gels were run under the same experimental conditions. Marker in kDa.

16E5 is predicted to encode three trans-membrane domains (TMDs), which were used as a basis to generate five previously described truncation mutants as GFP fusion proteins (Fig. 3A)^22^. The truncation mutants and FLAG-YIPF4 were expressed in HEK293T cells and a Co-IP performed using a FLAG antibody. The GFP-16E5 truncation mutants 1–83 (full-length), 1–79, 1–54 and Del1 (lane1, 2, 3, 6) clearly bound FLAG-YIPF4, only the 1–36 and 1–30 (Fig. 2B lane 4, 5) showed a substantial reduction in binding. The negative control Co-IPs confirmed the specificity of this assay (lane 7, 8). These results suggest that YIPF4 interacts with residues comprising the second TMD of E5.

**Figure 4:**
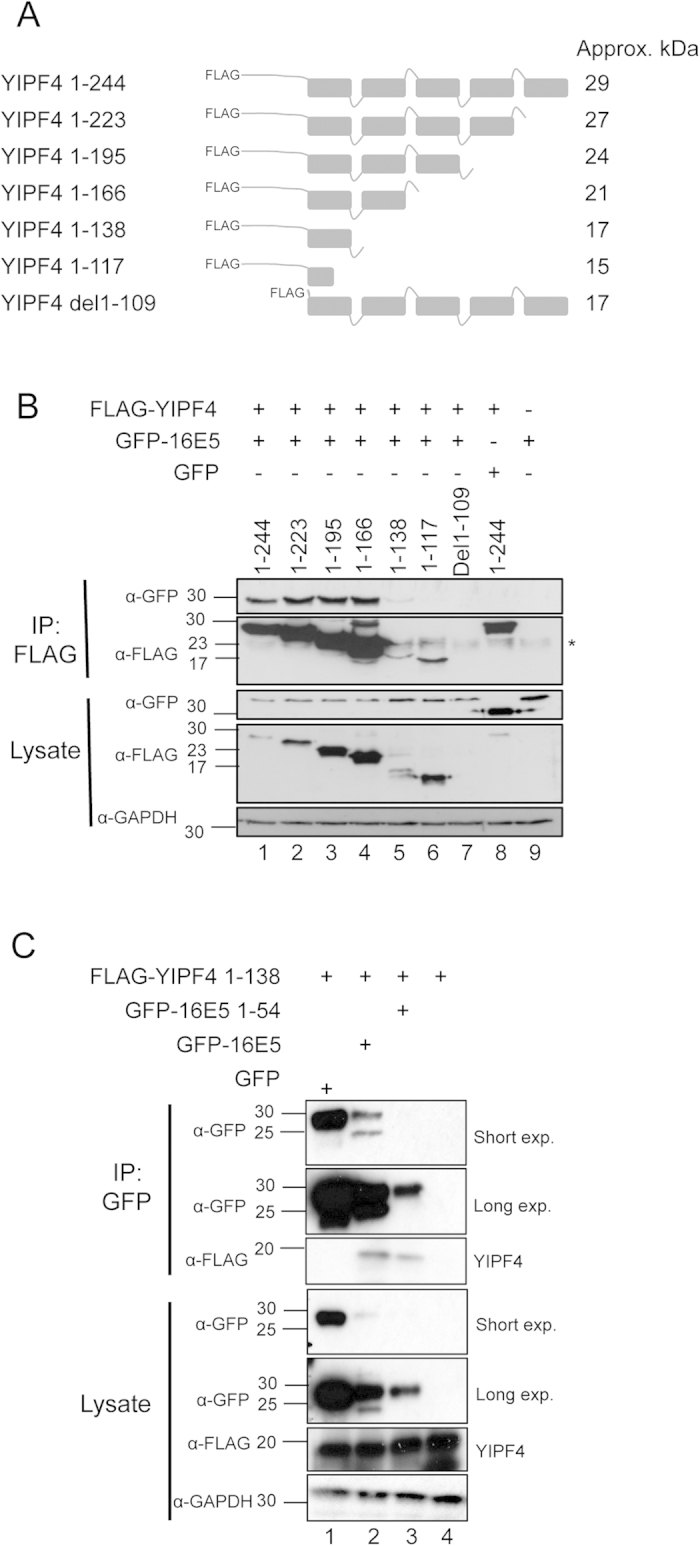
Mapping the regions of YIPF4 required to bind to 16E5. **A**) Schematic representation of FLAG-YIPF4 truncation mutants based on the five TMD model of YIPF4. **B**) Co-IP of FLAG-YIPF4 truncation mutants with GFP-16E5. Epitope tagged 16E5 and FLAG-YIPF4 truncation mutants were overexpressed in HEK293T cells. The del1-109 truncation mutant did not express. Co-IPs were preformed with equal amounts of total protein with FLAG antibody. **C**) Co-IP of minimal binding mutants of 16E5 and YIPF4. Lysates from HEK293T cells co-transfected with FLAG-YIPF4 1–138 and GFP, GFP-16E5 or GFP-16E5 1–54 were subject to GFP-TRAP precipitation. Precipitates were probed for GFP fusions and YIPF4 using the FLAG epitope. Lysates demonstrated equal loading of the FLAG-YIPF4, whilst E5 expression levels were significantly lower than GFP alone. All gels were run under the same experimental conditions. Representative blots are shown. Marker in kDa. * = antibody light chain.

We next sought to identify the region(s) of YIPF4 necessary for binding to 16E5. Computational models^23^ predict that YIPF4 possesses five putative TMDs. Truncation mutants were generated based on these predictions (Fig. 4A) with an amino-terminal FLAG epitope tag. The YIPF4 truncation mutants and GFP-16E5 were expressed in HEK293T cells and Co-IPs performed with FLAG antibody. The expression of 1-138 was low in comparison to other YIPF4 mutants and del1–109 was undetectable (Fig. 4B lanes 5, 7). The Co-IPs revealed that wild-type FLAG-YIPF4 (1–244), 1–223, 1–195 and 1–166 bound to GFP-16E5 (Fig. 4B lanes 1, 2, 3, 4). A faint band corresponding to GFP-16E5 was observed in the Co-IP with the 1–138 truncation mutant (lane 5). Only the 1–117 truncation mutant no longer bound by GFP-16E5 (Fig. 4B lane 6). Negative controls showed specific binding (Fig. 4B lane 8, 9). This approach highlights that residues within the amino terminal 138 amino acids may play a role in mediating the interaction with 16E5.

To fully validate that the regions within 16E5 and YIPF4 identified through our truncation analysis were sufficient to mediate the observed interaction, co-immunoprecipitations were performed using the YIPF4 1–138 truncation and GFP, GFP-16E5 or GFP-16 E5 1–54. As previously observed YIPF4 1–138 did not interact with GFP alone (Fig. 4C lane 1). Despite the considerably lower levels of fusion protein expression a clear interaction was observed with both full-length 16E5 and the 1–54 truncation (Fig. 4C lanes 2–3). Together these data indicate that the regions identified from the truncation screen are sufficient to mediate an interaction.

### YIPF4 is expressed in primary human keratinocytes

**Figure 5:**
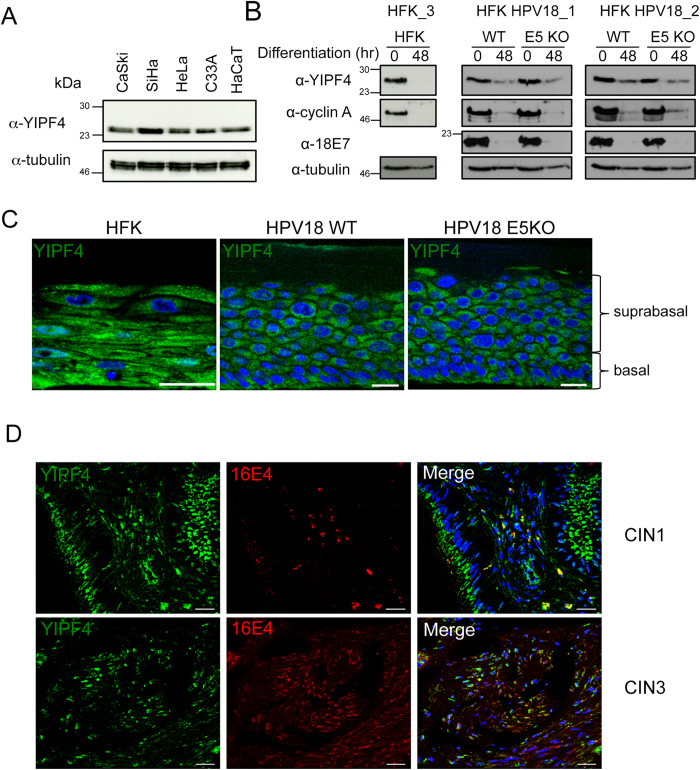
YIPF4 is expressed in primary keratinocytes and its expression is regulated by HPV. **A**) Panel of HPV positive (CaSki, SiHa, HeLa) and negative (C33A) cervical cell lines and the HPV negative skin cell line HaCaT probed by western blot using a YIPF4 antibody. Tubulin served as a loading control. **B**) Control and HPV18 harbouring HFK cells were differentiated in high calcium media for 48 hours and lysates probed for YIPF4 expression by western blot. Levels of YIPF4 protein decreased in NHK to greater levels than cells harbouring HPV18. Cyclin A and 18E7 served as host and virus markers of differentiation. All gels were run under the same experimental conditions. Markers in kDa. **C**) HFK cell lines were grown in organotypic raft cultures for 13 days. Histological sections were stained for endogenous YIPF4 (green). Representative images are shown. **D**) Pathological sections of HPV16 positive cervical intraepithelial neoplasia grade 1 and 3 were stained with YIPF4 (green) and 16E4 (red) antibodies. Cell nuclei were visualised with DAPI (blue). Scale bar = 20 μm.

To increase our understanding of the YIPF4 expression profile we screened a panel of established cell lines from the cervix (CaSki, SiHa, HeLa) and skin (HaCaT) using the commercially available YIPF4 antibody (Fig. 5A). YIPF4 expression was confirmed in all samples, showing a clear band of the expected molecular weight (27 kDa).

Since the HPV lifecycle is absolutely dependent on keratinocyte differentiation, we investigated YIPF4 protein expression in calcium differentiated primary human foreskin keratinocyte (HFK) cell lines. Untransfected HFK cell lines, (HFK) or transfected with WT (HFK HPV18_WT) or E5 knock out (KO) HPV18 genomes (HFK HPV18_E5 KO) were differentiated in media containing high concentrations of calcium for 48 hours. The decrease in HPV18 E7 and cyclin A levels after 48 hours confirmed differentiation of the cells (Fig. 5B). In untransfected HFK cells, the YIPF4 protein levels substantially decreased upon cell differentiation, however, YIPF4 expression was maintained in differentiated HPV18 WT and E5 KO positive cells to some extent indicating that the presence of the viral genome rescued YIPF4 expression levels – and this was independent of E5.

YIPF4 protein expression was also studied in organotypic raft cultures, a three dimensional cell culture model of stratified epithelium. Histological cross sections of organotypic raft cultures were examined for YIPF4 expression by immuno-histochemistry (Fig. 5C). Despite possible differences in YIPF4 expression in the basal layers of the epithelium YIPF4 protein appeared to be expressed throughout the epithelial layers in the raft cultures of HPV18 positive and negative cells lines implying that differences observed using calcium differentiation might not always translate into three dimensional raft cultures.

YIPF4 was also detected in clinical samples of pre-cancerous HPV16 positive cervical intraepithelial neoplastic (CIN) lesions of grade 1 and 3 (Fig. 5D). Co-staining of the samples with 16E4 confirmed that YIPF4 is expressed in sites of HPV16-induced pre-cancerous disease.

### Loss of YIPF4 does not affect HPV protein expression in undifferentiated cells

**Figure 6:**
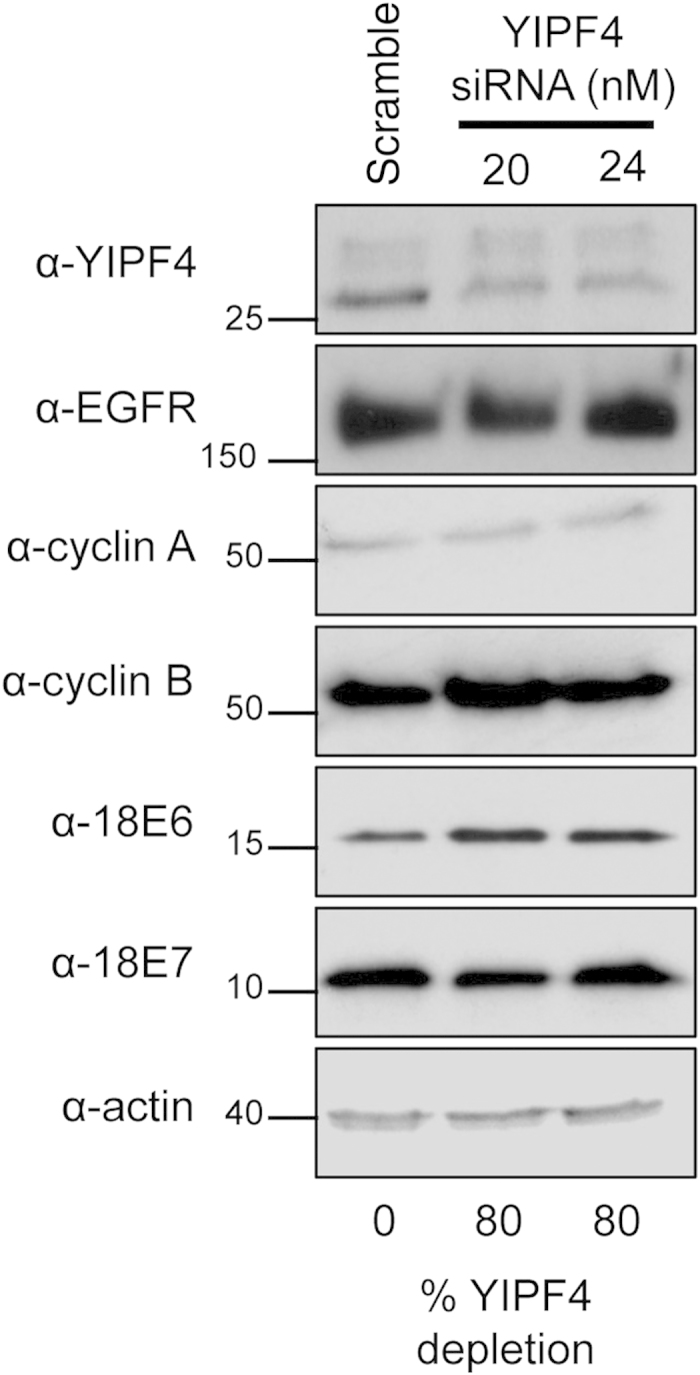
Depletion of YIPF4 does not impact on host and virus protein expression in undifferentiated primary foreskin keratinocytes containing HPV18. HFK harbouring the HPV18 genome were transfected with YIPF4 specific siRNA (20 nM and 24 nM) for 72 hours prior to cell lysis. Lysates were analyzed for host proteins associated with HPV infection including the growth factor receptor, EGFR, and markers of cell cycle cyclin A and cyclin B. Lysates were also probed for two HPV encoded early proteins, E6 and E7. Actin acted as a loading control. All gels were run under the same experimental conditions. Percentage of YIPF4 expression compared to scrambled control as determined by densitometry.

To assess whether depletion of YIPF4 would affect HPV protein expression primary human keratinocytes harbouring the HPV18 genome were transfected with siRNA aligning against bases 503–523 within the YIPF4 sequence and screened for expression of HPV early proteins, E6 and E7, and host proteins modulated by E5 including the EGFR and the cell cycle proteins cyclin A and cyclin B (Fig. 6). This approach achieved approximately 80% knockdown of the endogenous YIPF4 protein, as determined by densitometry, but had no consistent impact on the levels of the HPV or host proteins tested. These data suggest that YIPF4 expression is not required to maintain HPV early protein expression, nor does its absence impact upon the expression of growth factor receptor or cell cycle proteins tested.

### YIPF4 is not required for EGFR expression and recycling in HPV infected cells

**Figure 7:**
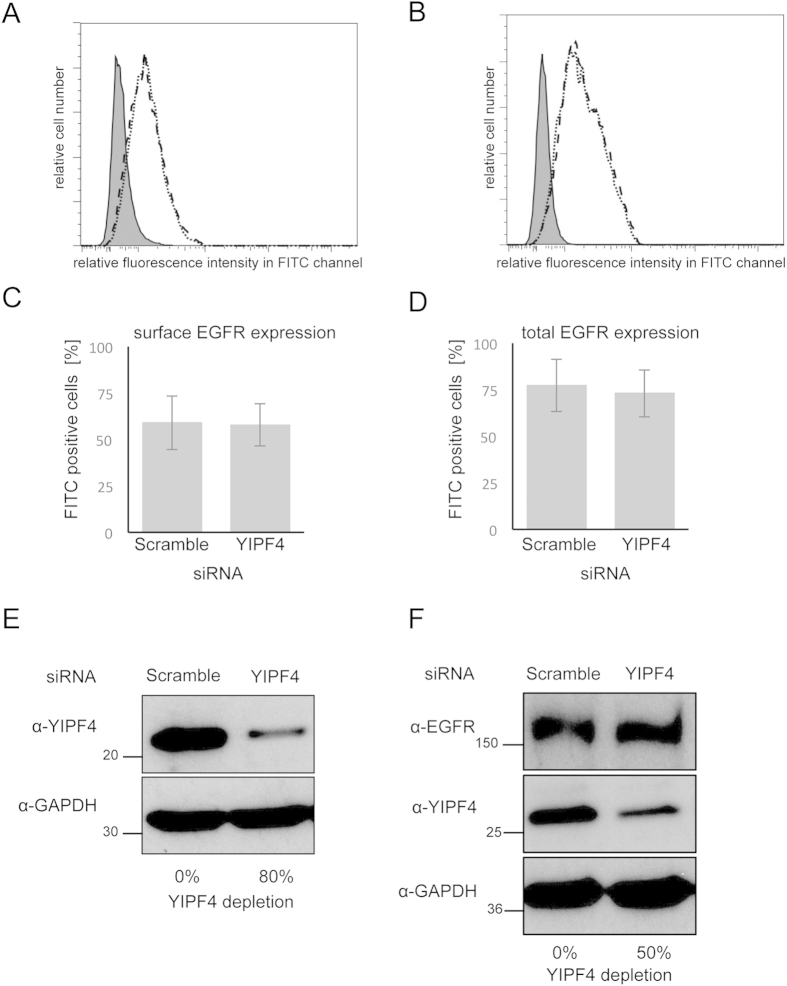
Total and surface EGFR expression in cells upon siRNA mediated knock down of YIPF4. C33A cells were transfected with scramble (control) or YIPF4 siRNA (24 nM). Cells were serum starved for 16 hours to analyse surface EGFR. 72 h post transfection cells were stained with EGFR and Alexa Fluor® 488 goat anti-mouse I gG antibody and subject to flow cytometry. **A**) Histograms of scramble and YIPF4 siRNA analysing surface EGFR expression. **B**) Histograms of scramble and YIPF4 siRNA analysing total EGFR expression. Filled histogram shows negative control, dotted line is control, dashed line is YIPF4. **C**) Graph representing the percentages of EGFR positive cells for the surface analysis. **D**) Graph representing the percentages of EGFR positive cells for the total analysis. **E**) Lysates from C33A cells transfected with scramble and YIPF4 siRNA were analysed for YIPF4 levels. Percentages of knockdown are shown. **F**) Primary keratinocytes containing the HPV18 genome were transfected with scramble and YIPF4 siRNA and differentiated for 48 hours in high calcium serum free media. Levels of total EGFR were analyzed by western blot. All gels were run under the same experimental conditions.

The transformative abilities of E5 have been linked to enhanced EGFR activity in a number of cell types^5^. In keratinocytes expressing wildtype HPV18 genomes a significant increase in both EGFR expression and activity has been observed and these are reduced in cells lacking E5 (Wasson *et al.*, unpublished data). Despite these observations, the molecular basis for the augmented EGFR activation is lacking. Since YIPF4 is implicated in pathways that intersect with EGFR trafficking we assessed whether YIPF4 was required for EGFR expression levels and surface localization in HPV negative and positive cells. YIPF4 protein was depleted from C33A cells using siRNA and total and cell surface localized EGFR determined using flow cytometry. No significant shift was observed in the histogram peaks between scrambled control siRNA and the YIPF4 specific siRNA for either surface localized or total EGFR (Fig. 7A–D), despite an approximate 80% reduction in YIPF4 protein levels as determined by densitometry (Fig. 7E). Levels of total EGFR were also assessed in primary keratinocytes harbouring the HPV18 genome. Silencing of YIPF4 resulted in an approximate 50% reduction in YIPF4 protein levels (Fig. 7F) but this did not translate into a noticeable loss in EGFR expression compared to a scrambled control. Together, these data suggest that YIPF4 is not required for EGFR expression or trafficking in cervical cells, nor is it co-opted by HPV to this pathway.

### YIPF4 does not regulate cell surface HLA class I expression

**Figure 8:**
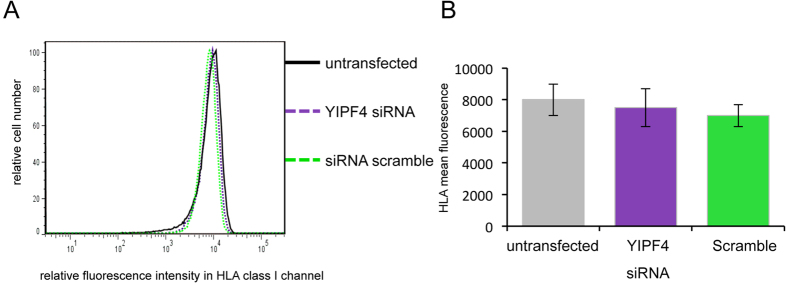
HLA class I cell surface expression in SiHa cells upon siRNA mediated knock down of YIPF4. SiHa cells were transfected with YIPF4 specific siRNA and a scambled siRNA control and stained with W6/32 and Alexa Fluor® 647 goat anti-mouse I gG antibody. Flow cytometry was used to analyse cells for their HLA class I cell surface expression. **A**) Histograms of a representative experiment are shown. **B**) The data from three independent experiments were analysed. A one-way ANOVA revealed no significant differences between the samples. Each bar represents the mean fluorescence of the HLA class I channel ± SDM.

E5 has been shown to deregulate the normal trafficking of host HLA class I molecules to the cell surface as part of an immune evasion strategy^8^. To determine whether YIPF4 plays a part in this process we investigated whether YIPF4 was required for the normal cell surface expression of HLA class I in uninfected cells. YIPF4 was depleted from SiHa cells using siRNA and surface HLA class I expression determined by flow cytometry (Fig. 8A). Quantification of the surface levels of HLA class I showed no significant differences between YIPF4 depleted cells and the untreated or scrambled controls (Fig. 8B). These data highlight that YIPF4 does not play a role in the normal cell surface trafficking of HLA class I proteins. Despite an attempt to study HLA class I surface expression in cells expressing E5, we were not able to find differences in HLA class I levels between control and E5 expressing cells (data not shown).

## Discussion

In this study, YIPF4 was identified as a novel HPV E5 interacting partner. YIPF4 (also known as FinGER4) is one of the nine members of the YIP1 domain family in humans^23^,^24^. It is a small integral membrane protein predicted to have five TMD, a hydrophilic N-terminus orientated onto the cytosolic face of membranes and its C-terminus buried within the membrane^23^,^25^. Endogenous YIPF4 resides mainly at the GA and might be involved in ER to GA trafficking or maintenance of the GA structure although the exact mechanisms have not been identified^19^,^26^. Since the family of Yip1 domain proteins is highly conserved^25^ it is conceivable that YIPF4 plays similar cellular roles as its orthologues in yeast. YIPF4 also forms complexes with its paralogue YIPF3^19^ but the role of these complexes remain to be elucidated. Here we show that 16E5 binds to YIPF4 by Y2H and Co-IPs. By the nature of the Y2H assay, it is likely that 16E5 binds directly to YIPF4 but it is not known whether these proteins are part of a larger complex possibly including the YIPF4 binding partner YIPF3.

We confirmed the presence of YIPF4 protein in primary human keratinocytes, the natural host cell of HPV infection. Upon differentiation, levels of YIPF4 protein reduced significantly and this reduction was lessened by the presence of HPV18. YIPF4 protein levels were also maintained in cells expressing a HPV18 genome lacking expression of the E5 protein, indicating that whatever mechanism was employed by HPV to prevent the decline in YIPF4, it is likely to be mediated by virus encoded proteins other than E5. In light of this, a bioinformatics analysis of the YIPF4 promoter revealed a number of possible E2 consensus sequences (data not shown). A concomitant decline in YIPF4 protein levels was not observed in organotypic raft cultures. This may reflect differences in the experimental systems or indicate that the immunofluorescence strategy to detect YIPF4 protein in raft cultures was not sensitive enough to reveal more subtle differences in expression levels during differentiation. Despite this there were possible differences in YIPF4 expression between HFK and HPV18 containing raft cultures, with potential changes in YIPF4 expression in the basal layers of the epithelium.

16E5 is predicted to have three trans-membrane α-helices with a luminal N-terminus and its C-terminus facing the cytosol^27^ which may build oligomers to form a viroporin^6^. Mutagenesis studies demonstrated that residues within the predicted second TMD of 16E5 likely mediated the interaction. This TMD mediates binding of E5 to the 16 K protein^28^. It is conceivable that YIPF4 and 16 K compete for 16E5 binding. BPV1 E5 interacts with the platelet-derived growth factor receptor-β as a dimer^29^, whereas it binds 16K subunit of the vacuolar H+-ATPase as a monomer^30^. 16E5 may thus exist in several oligomerization states in the cell, which exert different functions. YIPF4 is predicted to span the membrane five times although no published experimental work validates this model. We demonstrated the membrane association of YIPF4 and validated previous E5 studies^6^,^27^. Unlike E5, YIPF4 is not believed to form oligomers in cells^19^, although a potential dimer could be observed by western blot in some samples containing exogenous YIPF4. Preliminary mapping of the regions of YIPF4 required to bind to 16E5 implicated the amino terminus of the protein. However, these studies were hampered by poor expression of a number of the truncation mutants studied. In particular, a YIPF4 mutant lacking the predicted amino terminal hydrophilic region (del1–109) expressed to almost undetectable levels. Given the membrane-integrated nature of 16E5 and the preliminary binding assays from this study we predict that binding to 16E5 requires the first predicted TMD in YIPF4, rather than the hydrophilic region. However, a greater understanding of the regions of YIPF4 that contribute towards the interaction with E5 will require further mutagenesis of the amino terminal region. Our studies also demonstrate that YIPF4 is an interacting partner for all E5 proteins tested, including BPV E5. The interaction with E5 thus appears to be a potentially important function shared by all E5 proteins.

Despite the general paucity of information on the functions of E5, several of the known effects of E5 expression can be attributed to manipulation of intracellular trafficking. This is in accordance with the postulated functions of YIPF4. It is possible that the interaction with YIPF4 plays an important part in exerting functions such as the downregulation of HLA class I molecules to evade cellular immune response to HPV infection^16^,^22^,^31^ or the regulation of cell surface EGFR levels, which may promote transformation^32^. According to the literature, 16E5 promotes HLA class I molecules accumulation in the GA which then cannot be transported further to the cell surface^22^. Here we investigated whether the knock down of YIPF4 would affect the cell surface expression of HLA class I molecules. In this experimental system, the knock down of YIPF4 did not have an effect on the cell surface levels of HLA class I. It is still possible that loss of YIPF4 is compensated for by other members of the YIP1 protein family or by un-related proteins with redundant functions. YIPF4 might also be responsible for the trafficking of certain HLA class I types only. In this case the effect could have been concealed by the usage of the W6/32 antibody, which recognizes a wide variety of HLA class I types.

16E5 has also been shown to play a role in cell transformation by disturbing correct EGFR trafficking, which may be caused by deregulating the normal orchestration of receptor degradation processes and re-routing EGFR to recycling pathways. With the possible exception of the 16 K sub-unit of the vATPase, host proteins required for rewiring receptor trafficking are not known. As such it was plausible to investigate whether YIPF4 may contribute to this process. The observation that depletion of YIPF4 in both E5 positive and negative cells had negligible impact on either cell surface EGFR localization or total EGFR expression suggests that YIPF4 is unlikely to play a role in EGFR trafficking.

It has previously been reported that E5 aids in the maintenance of cell protein expression in infected keratinocytes^17^, and this correlates with increased mitogenic signaling in infected keratinocytes. In our hands, depletion of YIPF4 from keratinocytes harbouring the HPV18 genome had no impact on markers of cell cycle or on the expression levels of key early HPV proteins such as E6 and E7. These data therefore suggest that YIPF4 does not contribute towards regulation of the early stages of the HPV life cycle.

In conclusion, our study has identified a novel E5 interacting partner. We have mapped the regions of interaction for each partner and have demonstrated that YIPF4 is expressed in keratinocytes, where it localizes with E5 in the membraneous fraction of the cytoplasm. We demonstrate that YIPF4 is capable of binding to a range of E5 proteins, highlighting a potentially conserved role amongst these poorly studied proteins. Currently there is no defined function for the E5-YIPF4 interaction in either of the two better characterized roles for E5 in HLA class I and EGFR trafficking. Moreover, we also find no role for E5 in maintaining HPV protein expression or cell cycle expression in monolayer cells. However, we have only a limited number of parameters to test given the lack of understanding of E5 function and as such it is possible that it is important for a facet of the virus life cycle or pathogenesis that has yet to be elucidated. Further characterization of this complex will help to clarify the role of E5 proteins in HPV pathogenesis. This will be expedited by a more detailed knowledge of the basic cell biology of YIPF4 protein.

## Materials and Methods

### Cell culture

Transformed cell lines were cultured in Dulbecco’s modified Eagle medium (Lonza) supplemented with 10% Fetal Bovine Serum (GIBCO), 1 mM L-glutamine (Lonza) and 50 U/ml penicillin and streptomycin (Lonza), respectively. Human foreskin keratinocytes (HFK) were maintained in serum free keratinocyte medium (GIBCO). All HFK clones were maintained in E medium containing 2 mM L-glutamine (Lonza) and 5 ng/ml EGF (B.D. Biosciences) and were grown on mitomycin C (8 μg/ml, Roche) treated J2 3T3 fibroblast cells.

### HPV positive biopsy samples

Archival paraffin-embedded cervical biopsy samples were obtained with informed consent. Subsequent analysis of these samples was performed in accordance with approved guidelines, which were approved by Glasgow Royal Infirmary: RN04PC003. HPV presence was confirmed by PCR using GP5+/GP6+ primers.

### Generation of HPV18 genome containing HFK

For stably transfecting HFK with the HPV18 genome (HFK-HPV18), pGEM2-HPV18 (Sally Roberts, University of Birmingham, UK) was digested with EcoRI (NEB) to release the HPV18 genome, which was re-circularized using T4 ligase (NEB). The genome was co-transfected alongside pcDNA-Neo into HFK in serum free medium using Fugene6 (Roche). The cells were selected with 100 μg/ml G418 (Roche) E medium containing 5% serum, 2 mM L-glutamine, and 5 nM EGF for 8 days. Cells were selected on a layer of J2 3T3 fibroblast feeders (NIH) treated with 8 μg/ml mitomycin C (Roche). A stop codon was inserted after the start codon for E5 in the HPV18 genome to generate an E5 knock out genotype (HFK-HPV18E5KO) (Wasson *et al.* unpublished data).

### High Calcium Differentiation Assay

Keratinocytes were grown in E media until 90% confluent. Media was changed to serum free keratinocyte media containing 1.8 mM calcium chloride. Cells were maintained in this media for up to 96 hours before lysing.

### Organotypic Raft Cultures

Keratinocytes were grown in organotypic raft cultures by seeding the keratinocytes onto collagen beds containing J2 3T3 fibroblasts. Once confluent the collagen beds were transferred onto metal grids. The cells were allowed to stratify for 13 days before fixing with 4% paraformaldehyde (PFA).

### Construction of plasmid vectors

Plasmids containing 16E5 truncation mutations were obtained from Saveria Campo (University of Glasgow) in pcDNA-Neo with a haemagglutinin (HA) epitope at their N-terminus^33^. These were subcloned into the peGFP-C1 plasmid (provided by M. Hibma, University of Otago) excluding the HA epitope. The following HPV E5 sequences were cloned into peGFP-C2 using EcoRI and BamHI restriction sites: 2aE5 (isolate CN-HB1), 6bE5a^34^, 11E5a (isolate GUMC-AJ-Lung), 18E5 (isolate CU16) and 31E5 (isolate IN453545). The YIPF4 ORF was cloned into the HindIII and BamHI restriction sites of pcDNA3.1(+) fusing an N-terminal FLAG epitope tag. Truncation mutations were generated by inserting a stop codon after the indicated amino acid. Construction of the plasmid encoding the Cherry-FLAG-16E5 fusion protein was previously described^6^.

### Yeast two-hybrid screen (Y2H)

16E5 ORF was cloned by recombinatorial cloning (GATEWAY™, Invitrogen) into the bait vector pGBKT7. A semi-automated Y2H assay^35^ was used to screen against a HeLa cell and a human testis epithelial cell library in the pGADT7 prey vector (Clontech). Briefly, 16E5 bait was transformed into yeast strain AH109. Prey libraries in strain Y187 were mated with AH109 in yeast-peptone-dextrose-adenine media containing 20% polyethylene glycol 6000. They were divided equally into 96-well plates with interaction detection media (SD media lacking leucine, tryptophan and histidine, 5 μl/ml penicillin and streptomycin, 50 μM MuX and inhibitor 3-AT). Cells were plated on selective agar plates (SD media lacking Leucine and Tryptophan) to determine mating efficiency either by detecting individual colonies visually or by measuring fluorescence readout (excitation at 365 nm; emission at 448 nm). Positive single colonies were passaged once on agar plates (SD media lacking leucine, tryptophan and histidine) and prey cDNAs identified by PCR amplification and sequencing followed by RefSeq, ENSEMBL and Unigene databases.

### Transfections and mammalian cell lysis

Transient transfections were performed with a DNA to polyethyleneimine (PEI) ratio of 1:2 or a DNA to Lipofectin (Invitrogen, USA) ratio of 1:5. 24 h or 48 h post transfection, cells were lysed in lysis buffer for western blot or fixed for immuno-cytochemistry.

### Immuno-cytochemistry

Cells were grown on glass cover slips and transfected as described above. 24 h post-transfection, cells were fixed with 4% PFA for 10 min. Cells were washed in PBS and permeabilized with 0.1% Triton X-100 for 10 min. Non-specific targets were blocked by incubation in blocking buffer (0.2% gelatin, 10% normal goat serum (NGS), PBS) for 15 min. Cells were incubated with primary antibodies diluted in antibody solution (0.2% gelatin, PBS) for 1 hour. Cells were washed in PBS prior to incubation in secondary antibody in antibody solution for 1 hour. Cells were washed in PBS prior to mounting onto microscope slides using ProLong Gold Antifade Reagent with DAPI (Molecular Probes). Samples were observed under a Zeiss 700 laser scanning confocal microscope under an oil-immersion 63x objective lens (NA = 1.40).

### Immunohistochemistry

Paraffin embedded organotypic raft culture sections (5 μm) and clinical samples of HPV16 positive CIN1 and CIN3 lesions (kindly provided by S. Graham, University of Glasgow) were rehydrated and antigens were retrieved by 10 min boiling in sodium citrate buffer (10 mM Tri-sodium citrate, 0.05% Tween 20, pH 6). Sections were blocked (10% NGS, PBS) for 1 h and incubated with primary and secondary antibodies (1.5% NGS, PBS, 1h RT). Slides were mounted with DAPI containing agent (Invitrogen) and sealed. Primary antibody YIPF4 (Sigma) and 16E4 (kindly provided by S. Roberts, University of Birmingham) and secondary antibodies conjugated with Alexa 488 and Alexa 594 (Invitrogen) were used.

### Co-immunoprecipitation (Co-IP) and Western blotting

Co-IPs were performed using dynabeads protein G (Invitrogen, USA). The total protein content of the cell lysate was determined by BCA assay (Thermo Fisher Scientific, USA) according to the manufacturer’s protocol for microplates and the concentrations adjusted to the sample with the lowest concentration using binding buffer (50 mM Tris/Cl, pH 7.4; 100 mM KCl, 0.1 mM EDTA, 0.2% NP40, 0.1% BSA, 2.5% Glycerol, 2 mM dithiothreitol (DTT), 1 × Protease inhibitor cocktail, EDTA-free (Roche, Switzerland)). Magnetic dynabeads protein G slurry was equilibrated with binding buffer and was then incubated with respective antibody and a 1:1 mixture of adjusted cell lysate and binding buffer for 1 h at RT. After 4 washes with wash buffer (100 mM Tris/Cl, pH 7.4; 100 mM NaCl, 0.5% NP40, 2 mM DTT, 1 × Protease inhibitor cocktail, EDTA-free (Roche, Switzerland)), proteins were eluted by boiling for 10 min in 2 × LDS sample buffer (Invitrogen, USA) plus 0.1% 2-mercaptoethanol. All cell precipitates and lysates were resolved by SDS-PAGE and transferred to nitrocellulose membranes (Amersham Biosciences). Membranes were probed with antibodies specific for GFP (Clontech), YIPF4 (Sigma), FLAG (Sigma, EnoGene), TGN46 (kindly provided by S. Ponnambalam, University of Leeds) and GAPDH (Abcam). Blots were visualized using an ECL system (Amersham Biosciences).

### Subcellular fractionation

Cells were lysed in buffer M1 (10 mM PIPES, pH 7.4, 0.5 mM MgCl2, Protease Inhibitor Cocktail (Roche)) by sonication. Buffer M2 (10 mM PIPES pH 7.4, 600 mM KCl, 150 mM NaCl, 22.5 mM MgCl2) was added and samples were briefly centrifuged at 3000 xg for 10 min at 4 °C. The supernatant was subject to ultra-centrifugation at 100,000 xg for 10 min at 4 °C to pellet the membranous fraction. The pellet was washed once in adjusted buffer M1 and resuspended in sample buffer. The supernatant depicting the soluble fraction was subject to overnight acetone precipitation. Soluble fraction was resuspended in sample buffer and both fractions separated by SDS-PAGE and analyzed by Western blot as described above.

### Flow cytometry for cell surface HLA expression

Effective YIPF4 specific siRNA (20 nM and 24 nM) (aagaattggacattgatctaa, Qiagen) and siRNA scrambled control (24 nM) (Qiagen), respectively, were transfected with HiPerFect Transfection reagent (Qiagen) according to the manufacturers protocol. Cells were harvested, washed with PBS and blocked with 10% NGS in PBS for 1 h. Samples were labelled with HLA class I antibody (W6/32; 1:100; Abcam, UK) in antibody solution (1.5% NGS in PBS) for 1 hour. Cells were washed once in PBS before incubating with antibody solution and secondary Alexa Fluor® 647 goat anti-mouse immunoglobulin G (IgG) antibody (1:500, Invitrogen, USA; kindly provided by Prof G. Eric Blair) for 1 h. Samples were washed again and resuspended in PBS adding propidium iodide (PI; Invitrogen, USA) as a viability marker. Samples were analyzed on an LSRFortessa (BD Biosciences, USA) flow cytometer using the accompanied software (FACSDiva Version 6.2). All cell populations were gated to contain only live cells by excluding PI positive cells. For each sample, the mean fluorescence of the HLA class I detecting channel was determined using the FACSDiva software. The mean fluorescence values were compared by one-way ANOVA using OriginPro8.6 software. Histograms of fluorescence intensity in HLA/HLA-A201 class I detecting channel versus cell count were compiled with FlowJo software version 7.6.4 (TreeStar, USA) expressing the cell count as percentage of maximum cell count (=relative cell number).

### Flow cytometry for EGFR expression

Cells transfected with YIPF4 siRNA were fixed with 1% PFA and permeabilized with 0.1% Triton-X100 to assess total EGFR levels. Cells were serum starved 16 hours prior to fixation and not permeabilized to assess surface EGFR levels. Cells were stained with EGFR specific antibody (Santa Cruz) and an Alexa 488 conjugated mouse specific secondary antibody. Samples were analyzed on the LSRFortessa™ cell analyzer (BD).

## Additional Information

**How to cite this article**: Müller, M. *et al.* YIP1 family member 4 (YIPF4) is a novel cellular binding partner of the papillomavirus E5 proteins. *Sci. Rep.* **5**, 12523; doi: 10.1038/srep12523 (2015).
